# The AADR Visualizer: an ArcGIS online visualizer for ancient human DNA from the Allen Ancient DNA Resource

**DOI:** 10.1093/bioadv/vbaf199

**Published:** 2025-08-18

**Authors:** Woojung Yi, Elliot Delroba, Beth Zizzamia, Stephanie A Spera, Melinda A Yang

**Affiliations:** Department of Biology, University of Richmond, Richmond, VA 23173, United States; Department of Biology, University of Richmond, Richmond, VA 23173, United States; Department of Geography, Environment, and Sustainability, University of Richmond, Richmond, VA 23173, United States; Department of Geography, Environment, and Sustainability, University of Richmond, Richmond, VA 23173, United States; Department of Geography, Environment, and Sustainability, University of Richmond, Richmond, VA 23173, United States; Department of Biology, University of Richmond, Richmond, VA 23173, United States

## Abstract

**Motivation:**

The AADR Visualizer is designed to be a public, user-friendly, web-based graphical user interface for visualizing and filtering ancient humans included in the Allen Ancient DNA Resource (AADR), a regularly updated dataset that compiles published human genome-wide ancient DNA for research uses. This tool increases access to the rapidly growing pool of human ancient DNA available for study, facilitating genomic research and education focused on human prehistoric movement and interaction.

**Results:**

The AADR Visualizer provides the ability to (i) view all available individuals and associated metadata, (ii) filter individuals using a diverse set of geographic, temporal, and sequencing information, and (iii) search for individuals using group nomenclature as described in the Allen Ancient DNA Resource.

**Availability and implementation:**

The tool is freely available on the web at https://arcg.is/1CyL5n.

## 1 Introduction

The ability to retrieve and analyze genome-wide DNA from ancient humans has greatly enhanced our understanding of human genetic history. Sequencing of archaic hominins such as Neanderthals has revealed widespread archaic ancestry in modern humans (Green *et al.* 2010, Reich *et al.* 2010, Meyer *et al.* 2012, Prüfer *et al.* 2014, Fu *et al.* 2015). Extensive sampling across western and central Eurasia has uncovered three layers of ancestry in present-day Europeans associated with prehistoric hunter-gatherers indigenous to the region, early farmers from the Near East, and steppe pastoralists from Central Asia (Haak *et al.* 2015, Lazaridis *et al.* 2016). Retrieval of ancient DNA (aDNA) from humans across Asia and Siberia reveal a diverse genetic landscape before the Holocene that differentially impacted present-day Asian and Native American populations (Fu *et al.* 2013, Raghavan *et al.* 2014, Yang *et al.* 2017, Lipson *et al.* 2018, McColl *et al.* 2018, Wang *et al.* 2021). Importantly, rapid improvements in aDNA extraction and sequencing technology over the last fifteen years (Fu *et al.* 2013, Haak *et al.* 2015, Mathieson *et al.* 2015, Rohland *et al.* 2015, Slon *et al.* 2017) have led to the sequencing of over 10 000 ancient humans, from all populated continents and as far back as 50 000 years ago.

As the collection of human aDNA has grown, so too have efforts to improve access to these data. The Allen Ancient DNA Resource (AADR) (Mallick *et al.* 2024) was developed to be a regularly updated, commonly formatted compendium of all published human genome-wide aDNA for the 1 233 013 single nucleotide polymorphisms (SNPs) that have been targeted for retrieval across most human aDNA studies. First shared online in 2019 and published in 2024, the AADR has been widely accessed for ancient human DNA research, with over 20 000 downloads and over 60 citations within the first year of publication alone (Mallick and Reich 2024).

Despite the strides that the AADR has made for data accessibility, it is still difficult to rapidly examine the available human aDNA and determine appropriate individuals for inclusion in new research studies. When and where the individual lived impacts whether their inclusion will address a proposed research question; and the sequencing method and amount of DNA successfully sequenced may impact which analyses are appropriate. Moreover, because familial relationships can bias most demographic analyses, related individuals often need to be excluded. With the large number of ancient individuals sequenced to date, then, determining who to include in analyses for a research question is nontrivial. And although a major feature of the AADR is that it contains a vast repository of metadata for each sample, scanning and filtering these metadata requires proficiency in a computational language.

Though efforts to improve access to the AADR exist (Harris and Greenbaum 2024), the results limit the metadata available and excludes grouping nomenclature (Eisenmann *et al.* 2018) that is necessary when deciding whether or not to combine individual genomic data to mitigate the impact of missing data and allow the incorporation of frequency data for population genetic analyses. Therefore, there exists a need for a tool that not only displays the temporal and spatial features of available individuals, but also integrates grouping nomenclature and other metrics, improving access to human aDNA in future studies.

Here, we present a public-facing, user-friendly ArcGIS Online graphical user interface (GUI), the AADR Visualizer (https://arcg.is/1CyL5n). This visualizer includes all ancient humans from the AADR and their associated metadata. Below, we describe the features associated with the AADR Visualizer, including (i) the ability to view and download extensive metadata for ancient individuals, (ii) a large and diverse set of filtering tools, and (iii) the ability to locate sets of individuals using grouping nomenclature (Group ID) as designated in the AADR.

## 2 Methods

### 2.1 Allen Ancient DNA Resource (AADR) metadata ETL Python pipeline

The AADR Dataverse 9.0 (v62.0, 16 September 2024) includes 13 571 human aDNA samples and 4054 present-day samples from the 1000 Genomes Project (Auton *et al.* 2015), 929 Diverse Genomes Project (Bergström *et al.* 2020), and Simons Genome Diversity Project (Mallick *et al.* 2016), among others—all mapped against the hg19 reference genome (Lander *et al.* 2001, Schneider *et al.* 2017, Fairley *et al.* 2020). Since the objective of the AADR is to document and store genetic material for ancient humans, and the 4054 present-day humans are well documented elsewhere and included in the dataset primarily for facilitating demographic analysis, we did not include them in the AADR Visualizer.

The AADR includes a file where each row contains the associated metadata for a unique genetic sample (genetic ID, Table 1), and each column is a type of metadata (e.g. latitude, longitude, coverage, file extension: *anno*). Note that we use the terms sample and individual interchangeably, though there may be a small number of samples that derive from the same individual, indicated by a matching master ID (Table 1). A total of 35 columns are present, including dating information, associated geographic location (e.g. latitude, longitude, locality), grouping nomenclature (i.e. Group ID), sequencing information and metrics (e.g. experimental methods, number of autosomal SNPs), and other identifying information (e.g. molecular sex, mtDNA and/or Y-chromosome haplogroups, associated publication). We subset that dataset to extract 22 metadata columns, focused on information related to dating, location, sequencing metrics and process, and identifying information (Table 1). For any missing or conflicting data, our approach differed depending on the metadata type, as described below.

**Table 1.** List of downloadable metadata from the AADR Visualizer.

Metadata	Corresponding header in AADR	Description of metadata
genID	Genetic ID	Unique ID assigned to each DNA sample in the AADR
masterID	Master ID	Identifier for data from the same individual
groupID	Group ID	Group name assigned to a set of geographically proximal individuals with similar genetic characteristics
publication	Publication abbreviation	AADR publication abbreviations
doi	doi for publication of this representation of the data	Digital Object Identifier provided by AADR
ybp	Date mean in BP in years before 1950 CE	Average dating, OxCal mu for a direct radiocarbon date, and average of range for a contextual date
yrange	Full Date One of two formats.	Description of full date range, in one of two formats: (i) 95.4% CI calibrated radiocarbon age (Conventional Radiocarbon Age BP, Lab number) or (ii) Archaeological context range
locality	Locality	Area of country where individual was buried
political_entity	Political Entity	Country provided by AADR
lat	Lat.	Latitude provided by AADR
lon	Long.	Longitude provided by AADR
snpauto	SNPs hit on autosomal targets	# of autosomal SNPs available for sample, computed using easystats on 1240k snpset
molsex	Molecular Sex	AADR calculated sex of individual, based on genetic data
yhaplo_term	Y haplogroup in terminal mutation notation	AADR calculated, automatically called based on Yfull with published software (Lazaridis *et al*. 2022)
yhaplo_isogg	Y haplogroup in ISOGG v15.73 notation	AADR calculated, automatically called based on Yfull with published software (Lazaridis *et al*. 2022)
mtDNA_covg	mtDNA coverage (merged data)	AADR calculated coverage of mtDNA in the merged data
mtDNA_haplo	mtDNA haplogroup if >2× or published	AADR assigned haplogroup for individuals with >2× mtDNA coverage
dmgrate	Damage rate	AADR calculated for first nucleotide on sequences overlapping 1240k targets in the merged data
libtype	Library type	Library development, including amount of damage correction and double-stranded or single-stranded library prep
asm	ASSESSMENT	AADR provided assessment of data validity
repository	Link to the most permanent repository hosting these data	AADR provided accession number or link to repository where original data are located
sequence_type	Suffices (indicating data types used for sources which can be a subset of that in bam)	AADR provided information on the type of sequencing method used to generate the raw data for the provided sample
GISLat	N/A	AADR provided and manually determined latitudes used for mapping in Visualizer
GISLon	N/A	AADR provided and manually determined longitudes used for mapping in Visualizer
region	N/A	ISO-3166 regions associated with countries
sub-region	N/A	ISO-3166 sub-regions associated with countries
doi_link	N/A	Link to original publication associated with sampled individual
notes	N/A	Description of manual edits done, blank if no edits were performed

To highlight the spatial variability across samples in the AADR Visualizer, geographic coordinates (i.e. latitude and longitude) were required to include each individual on an online map. For each entry missing geographic coordinates, we examined the corresponding publication to determine if a set of coordinates was available. If unavailable, we then looked through the figures in the corresponding publication showing the sampling location and approximated the coordinates on the map. As a last resort, we used available information in other metadata (e.g. locality, group ID) to approximate the general region and assign latitudes and longitudes. New coordinates and the method used to determine them were indicated in an additional column noting manual edits (notes, Table 1). The coordinates already available in the AADR and the newly assigned coordinates were used to populate two new columns (GISLat, GISLon, Table 1). GISLat and GISLon were then used to map each individual. Political entities listed in the AADR were matched to a standardized list of countries (ISO-3166, v10.0, https://github.com/lukes/ISO-3166-Countries-with-Regional-Codes.git), with associated country codes, regions, and sub-regions. For individuals assigned to a country in a different region than where they are located (e.g. St Martin’s is assigned to the Netherlands but is in the Caribbean), we manually edited the region and sub-region to match their location.

The AADR includes a list of publication reference codes and digital object identifiers (DOI) linking each entry to a single publication, typically the most recently published study, as that publication would include the best quality version of the genome-wide data from that individual (Mallick *et al.* 2024). We obtained and verified hyperlinks associated with each publication for inclusion in the visualizer. Any missing DOIs were manually retrieved and included. Again, when coordinates or DOIs were not available or required editing, and when regions/subregions were assigned manually, a description of the manual edit was added for that individual. All extracted and new metadata for each ancient individual were then exported into a CSV file.

### 2.2 ArcGIS online dashboard and experience website development

The resulting CSV was uploaded to ArcGIS Online as a hosted feature layer. Each row of data from the CSV table was plotted on a web map using the newly created GISLat and GISLon coordinates as a point feature. The web map was designed to default display at a global scale, where clustering symbology is used to aggregate points by geographically proximal samples to create a clean map view. As a user zooms in, the points automatically adjust to represent smaller clusters, as noted by the number and size of the symbol. At the greatest magnification, each point would represent one or more individuals with the same coordinates. Note that the symbol size is scale dependent, where the size of the circle and numerical label indicates the number of individuals in that cluster, and the color of that circle indicates the average number of autosomal SNPs available across those individuals, with a dark blue point indicating that the individuals in the cluster contain an average of <100 000 autosomal SNPs, and a brown point indicating that the individuals in the cluster contain an average of more than 500 000 autosomal SNPs.

The web map was customized so that when a user clicks on a point, a customized pop-up window appears. For a point representing a single individual, the pop-up shows metadata for that individual. For a clustered point representing multiple individuals, the pop-up shows a “cluster summary,” which is a summary of select metadata, along with a list of Group IDs and the number of individuals associated with each Group ID. For cluster summaries, visualizing metadata specific to each individual was also made available in the pop-up through the “Browse features” tab.

The resulting web map was embedded in an ArcGIS Dashboard to allow for additional functionality, including allowing the user to filter individuals based on various parameters. The user can use a sliding scale to filter the data based on radiocarbon dating (Years) and the number of autosomal SNPs available. The data can also be filtered by country (Political Entity), region, and subregion–categorical data that were derived from a standardized set of options based on the ISO-3166 country list described earlier. A Group ID Search function allows the user to choose from a drop-down list of groups associated with a particular country. Additional options include filtering by sequencing type (e.g. Twist capture, whole genome sequencing) or uniparental haplogroups. Lastly, the AADR Visualizer includes a linked attribute table that updates as a user applies filters, and the updated attribute table can be downloaded by the user.

This dashboard was then embedded in an ArcGIS Experience. Where a dashboard increases the functionality of a web map, an ArcGIS Experience can be thought of as a website that houses the dashboard and any other additional information on different pages. We added an information page including instructions for use and explanations of symbols used by the AADR and a feedback form to assist in improving usage in future versions.

## 3 Results and discussion

Here, we present the AADR Visualizer (v1.1, https://arcg.is/1CyL5n), an ArcGIS Online interactive GUI that allows for intuitive, fast, and convenient visualization of ancient human genome-wide data from the Allen Ancient DNA Resource, a regularly updated archive of published genome-wide aDNA (Mallick *et al.* 2024, Mallick and Reich 2024). The main feature is an interactive map including all 13 571 ancient humans from AADR Dataverse 9.0 (v62.0, September 16, 2024), which can be easily updated to incorporate new version updates from the AADR.

**Figure 1.**
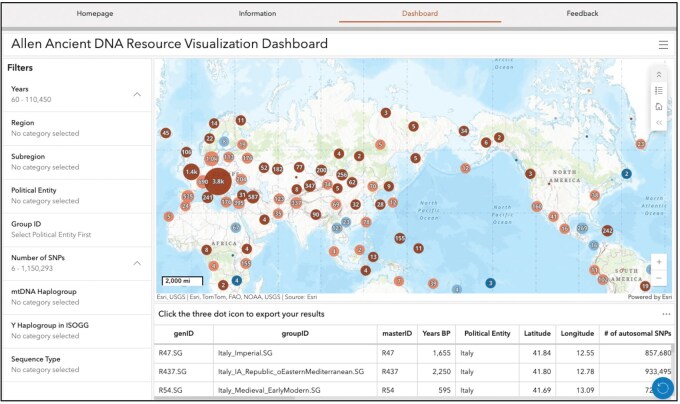
Visualization of ancient individuals from the Allen Ancient DNA Resource (AADR) in the AADR Visualizer. Center, world map on which AADR individuals are mapped. Points indicate aggregated individuals (i.e. clusters) from the AADR due to their geographic proximity to each other, with the numeric label and size of the point indicating the number of individuals included in the cluster. Colors indicate the average number of autosomal SNPs available for individuals in the cluster (>500k: brown, 300–500k: peach, 100–300k: light blue, <100k: dark blue). Zooming, filtering, and moving the map dynamically adjusts the number of entries included in a cluster. Left, list of categories for filtering data visualized on map. Below, a downloadable table including all individuals displayed on the map that dynamically responds to both zooming and filtering. The blue circle in the bottom right resets the visualizer to defaults.

We noted above that live clustering symbology was used to group data points that are in close geographic proximity relative to the zoom scale of the map. For example, the western coast of North America shows a medium sized point with the number 160 (Fig. 1). This indicates that there are 160 individuals that plot to this general region. Upon zooming in, the data becomes multiple points with updated symbology to indicate the number of individuals at smaller geographic scales.

**Figure 2.**
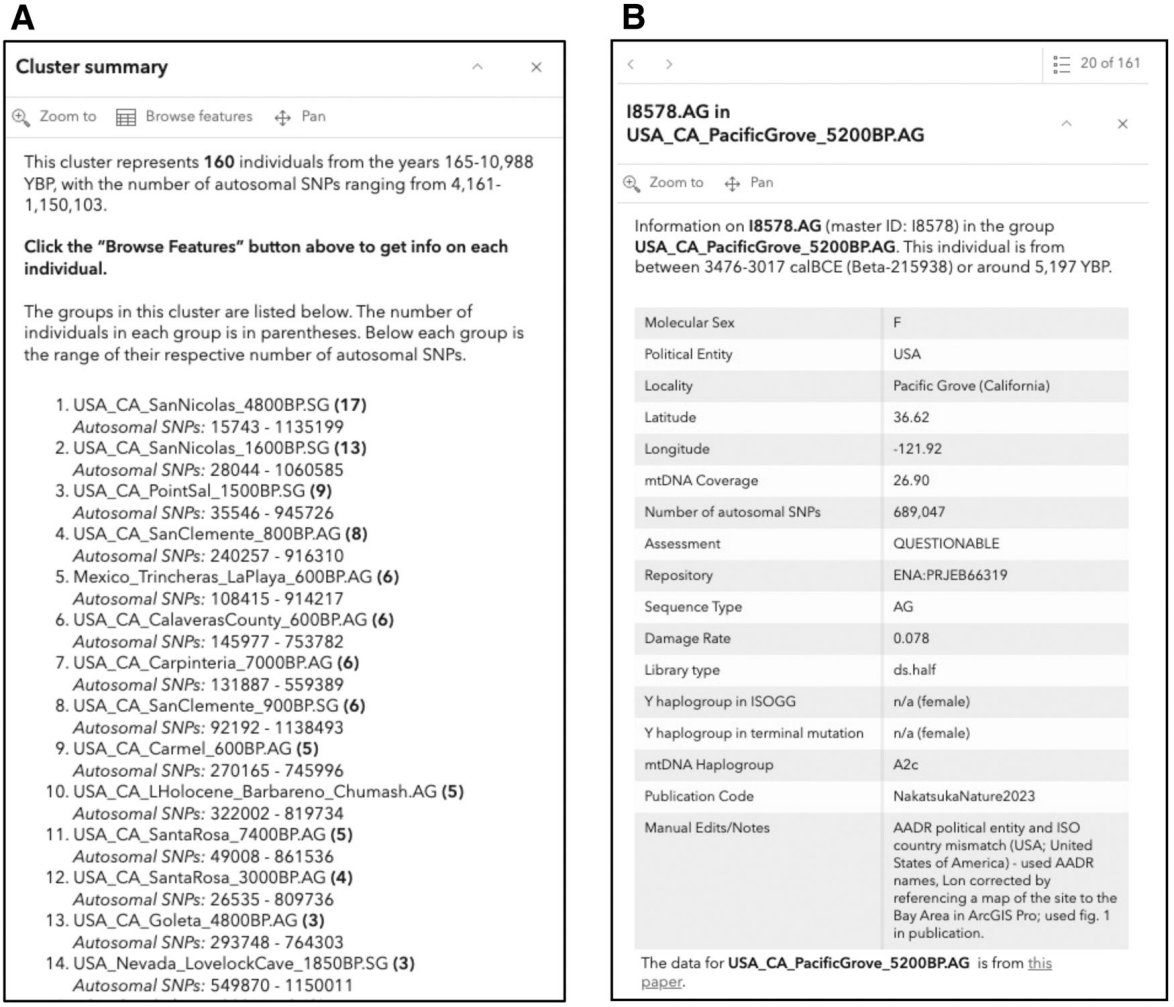
Example of visualization in pop-ups associated with clusters and samples available on the AADR Visualizer. (A) Pop-up showing a “cluster summary,” or information for groups found in a cluster, with the number of samples per group indicated in parenthesis. The example shows 14 of the 67 groups representing 160 samples in North America (circle with the number 160 in Fig. 1). Clicking “Browse features” at the top of the pop-up window will allow navigation to unique individuals, designated by their unique Genetic ID. (B) Pop-up for a single ancient individual, I8578 from Pacific Grove, CA, USA. Metadata is visualized in the pop-up in three locations. Identifier and dating information are summarized in a paragraph at the top. Geographic, sequencing, and molecular information are summarized in a table, along with any Manual Edits performed on the AADR metadata. No text indicates no manual edits were performed. A hyperlink to the associated publication (Nakatsuka *et al.* 2023) is available below the table.

Summary statistics about the individuals contained in that cluster can be viewed in a pop-up window that opens when you click on each point (Fig. 2A). A detailed description of the metadata associated with the individuals included in each cluster can be examined by clicking “Browse features” at the top of the pop-up (Fig. 2). For each individual, identifier and dating information is described at the top of the pop-up, a hyperlink to the corresponding publication is provided at the bottom, and a table of geographic, sequencing, and molecular information, including permanent repository information for the original data, is included in an embedded table (Fig. 2B).

There are numerous filtering options to quickly visualize individuals based on preferred metrics. These include filtering by time, geography, sequencing method, number of autosomal SNPs, and uniparental haplogroups through the ArcGIS Dashboard (left sidebar, Fig. 1). Filters are fast and responsive, making it easy to find individuals of interest on the AADR Visualizer. An additional filter for Group ID is available; however, due to the large number of unique categorical data associated with the Group ID field, the initial responsiveness of the AADR Visualizer lagged. To mitigate the slow speed of the Group ID filter tool, we provided instructions to the user that Group IDs are best filtered after first subsetting to a set of specified countries using the Political Entity filter. Once a set of preferred individuals are visualized on the map using filter or zoom tools, the corresponding metadata for all of those individuals can be immediately downloaded as a CSV file for further use (Data 1, available as supplementary data at *Bioinformatics Advances* online). Collectively, these features allow the user to quickly search and visualize human aDNA availability and metadata that fit their preferred search parameters.

Across the AADR dataset, the location and DOIs for 60 individuals were manually edited. Twenty-seven individuals were missing DOIs, which we included by searching for the associated publication. Thirty-three individuals were missing coordinates, which were added to the GISLat and GISLon columns using the methods described above and annotated in the “notes” column. A comparison of ArcGIS Online country borders to coordinates indicates that 146 individuals were mapped in a water body and another 56 were mapped to a different country than provided in the metadata. After examining the samples, we concluded that five individuals (I11552, I21452, I21249, I21250, and I8578—shown in Fig. 2B) had typos in the coordinates in the original publication so we edited the coordinates by referencing other samples in the original publication (Nakatsuka *et al.* 2023). All those mapped in a water body were in the assigned country, and all others were geographically close to the assigned country, so conservatively, we did not adjust these samples’ coordinates.

To date, only one other visualizer—Data Overlays for Research in Archaeogenomics (DORA) (Harris and Greenbaum 2024)—has been developed. The AADR Visualizer differs from DORA in that the AADR Visualizer has more comprehensive filtering capabilities. Uniquely, the AADR Visualizer emphasizes Group IDs, allowing the user to examine individuals by standardized grouping nomenclature provided by the AADR. This feature is useful as many population genetic tools for analyzing human aDNA (e.g. Admixtools) (Patterson *et al.* 2012) rely on grouping genetically similar individuals to increase statistical power for inferring demographic history and genetic relationships. Thus, identifying the number of groups available for a particular region or time period is often more useful than just identifying the number of individuals. A filter tool based on Group IDs that allows for quick identification of ancient human groups is especially useful in regions where dense sampling has been performed.

The AADR Visualizer features intuitive visualization and facilitates data exploration through the live clustering symbology that quickly provides summary statistics and comprehensive metadata for individuals in the corresponding pop-ups. Unlike DORA, which can only show metadata for one individual per location in its pop-ups, the AADR Visualizer shows metadata for all individuals from the same coordinates directly in its pop-up windows. Moreover, the AADR Visualizer’s downloadable table is not only filter- and map-responsive, but it also includes more of the original metadata associated with each individual, including uniparental haplogroups and full radiocarbon dating. While the AADR Visualizer does not have genetic analyses like DORA (Harris and Greenbaum 2024), the reduced tool complexity allows for more convenient and accessible exploration of available ancient human genome-wide data.

The AADR Visualizer will be regularly updated with new AADR releases, which typically occur every few months (Mallick and Reich 2024). With each new AADR release, the updated metadata will be downloaded and processed using the AADR Metadata ETL Python Pipeline described above, with manual edits incorporated for flagged samples where needed. ArcGIS Online can easily incorporate new entries, revisions to old entries, and the addition of new fields so long as a unique identifier (i.e. Genetic ID) remains the same across versions. User feedback will be reviewed with new AADR updates or every four months, whichever is sooner. With each new update, a description of edits made to the AADR Visualizer will be shared on the homepage.

We intend for this visualizer to lower the barrier for researchers interested in incorporating human aDNA into their studies. As the number of ancient humans sampled exponentially increases with improved sequencing techniques and the AADR dataset grows, understanding who these ancient humans are, and which of these humans may be relevant to a particular research question is integral. Visualizing the data on an interactive map, allowing for multiple filtering options especially by Group ID, and providing access to crucial sample metadata will help users sift through available human aDNA across the globe and thus take full advantage of the AADR’s compilation of published genome-wide data. Moreover, we hope that these features and the web-based GUI will also help overcome barriers related to computational skills and can be used as a teaching tool in undergraduate classrooms to explore publicly available human aDNA. While accessibility of the population genetic tools used in studying ancient humans is still limited, the AADR Visualizer is a first step in expanding access to these data for all.

## Supplementary Material

vbaf199_Supplementary_Data

## Data Availability

The data underlying this article are available in the Allen Ancient DNA Resource (Dataverse 9.0) (Mallick *et al.* 2024, Mallick and Reich 2024) at https://doi.org/10.7910/DVN/FFIDCW.

## Web resources

The AADR Visualizer (v1.1) is available at https://arcg.is/1CyL5n, and information on how to use the Dashboard and any future version updates will be found at this link. The Python ETL pipeline described in this study can be found at https://github.com/MYangLab/aadr-visualizer.
